# Is it Necessary to Evaluate Fear of Childbirth in Pregnant Women? A Scoping Review

**DOI:** 10.1055/s-0042-1751062

**Published:** 2022-06-29

**Authors:** Cibele Santini Oliveira Imakawa, Mariane Nunes Nadai, Monica Reis, Silvana Maria Quintana, Elaine Christine Dantas Moises

**Affiliations:** 1 Faculdade de Medicina de Ribeirão Preto, Universidade de São Paulo, Ribeirão Preto, SP, Brazil; 2 Faculdade de Odontologia de Bauru, Universidade de São Paulo, Bauru, SP Brazil; 3 Pan American Health Organization, World Health Organization, Washington, United States; 4 Department of Gynecology and Obstetrics, Faculdade de Medicina de Ribeirão Preto, Universidade de São Paulo, Ribeirão Preto, SP, Brazil

**Keywords:** fear, childbirth, obstetric labor, pregnant, delivery, medo, parto, trabalho de parto, gestante, via de parto

## Abstract

**Objective** To review concepts, definitions, and findings about fear of childbirth (FOC).

**Methods** A bibliographic review was carried out through the main scientific databases in 2020.

**Results** All 32 articles considered potentially relevant were analyzed. A recent study suggests that the global prevalence of FOC can reach up to 14%. Factors such as parity, gestational age, previous birth experience, age and nationality of the woman seem to influence FOC.

**Conclusion** Fear of childbirth could be related to an increased risk of adverse obstetric outcomes such as maternal request for cesarean delivery, preterm birth, prolonged labor, postpartum depression, and post-traumatic stress. These evidence highlight the importance of the discussion regarding this topic.

## Resumo

**Objetivo** Revisar conceitos, definições e achados sobre medo do parto (MDP).

**Métodos** Foi realizada uma revisão bibliográfica nas principais bases de dados científicas em 2020.

**Resultados** Foram analisados todos os 32 artigos considerados potencialmente relevantes. Um estudo recente sugere que a prevalência global do MDP pode chegar a 14%. Fatores como paridade, idade gestacional, experiência anterior de parto, idade da mulher e nacionalidade parecem influenciar o MDC.

**Conclusão** O MDC pode estar relacionado ao aumento do risco de desfechos obstétricos adversos, como solicitação materna de cesariana, parto prematuro, trabalho de parto prolongado, depressão pós-parto e estresse pós-traumático. Estas evidências destacam a importância da discussão sobre este tema.

## Introduction

The expression "fear of childbirth” (FOC) could have a substantial impact on the choice of delivery mode and, therefore, on maternal-fetal outcomes. Fear is a primary and basic emotion within a spectrum that comprises concerns, varying in intensity from mild and strong fear to phobia. 1 There is no consensus in the literature regarding the definition of FOC. This is a broad concept, and it is used to describe the types of anxiety and fears experienced by women regarding pregnancy and childbirth. 2 Within this context, there are different denominations used, and there is no standardization of appropriate assessment tools of FOC. 3 Thus, FOC represents an extensive area for research, with many gaps regarding multiple aspects of this topic still needing to be filled. 2 Thus, the aim of the present review is to review concepts, definitions, and findings about FOC, to contextualize the importance of its discussion during prenatal care, and, therefore, contextualize the importance of its discussion during prenatal care.

## Methods

The topic of FOC has many gaps, from its definition to its diagnosis and evaluation. Due to the relevance of this theme in clinical practice, a narrative review was carried out to bring up some central aspects on this subject and, thus, encourage the investigation of FOC during prenatal care. Therefore, a comprehensive bibliographic review was carried out through an electronic search dating from January 2000 up to December 2020, based on the recommendations set out in the Preferred Reporting Items for Systematic Reviews and Meta-analyses (PRISMA) statement, in the following databases: PubMed, MEDLINE, Cochrane Library, LILACS, and SciELO. The search was made regarding the definition of TOC and its evaluation using the following search terms: *pregnant women* AND/OR *pregnancy* AND *fear of birth* AND/OR *fear of childbirth* , based on Health Sciences Descriptors (DeCS) and Medical Subject Headings (MeSH). The search was initially restricted to studies published in Portuguese or English, performed on humans, review articles (systematic review and/or meta-analysis), clinical trials (randomized or not), and clinical protocols. When no clinical trials were found for the topic sought, the search for observational studies was included.

## Results

**Fig. 1.**
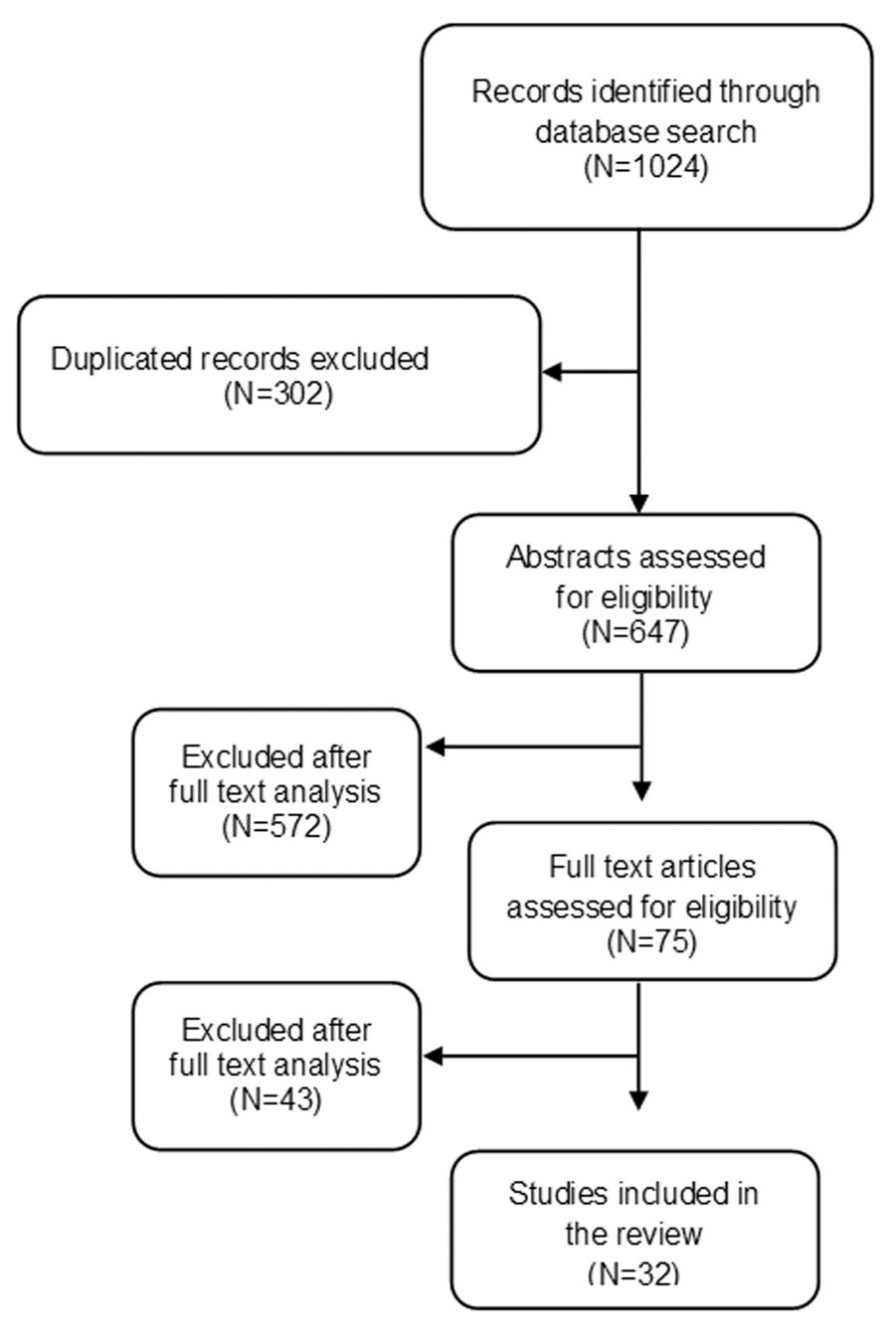
Flowchart of the study.

The searches yielded 1,024 articles, 302 of which were excluded because they were duplicates in the databases. A total of 572 articles were excluded after the analysis of the titles and abstracts, and 44 were excluded after full text analysis because they failed to meet the study objectives. After the first evaluation, 31 full texts of articles considered potentially relevant were retrieved and analyzed in detail ( Figure 1 ).

The reference lists of all full articles retrieved were analyzed to identify other potentially relevant articles from the title. The main findings are reported in the discussion session ( Table 1 ).

**Table 1:** Main findings of the selected studies

Title	Country, year	Outlining/ number of patients	Interventions	Outcomes/Results
Psychological perspectives on fear of childbirth 1	Sweden, 2016	Narrative review, 86 studies	N/A	To examine the literature on FOC from a psychological perspective
Definitions, measurements and prevalence of fear of childbirth: a systematic review. 2	Sweden, 2018	Systematic review, 24 studies	N/A	To summarize published research on the prevalence of FOC in childbearing women and how it is defined and measured during pregnancy and the postpartum period
Potential mechanisms in fear of birth: The role of pain catastrophizing and intolerance of uncertainty. 3	Sweden, 2019	Cross-sectional study, 499 women, FOBS	N/A	To investigate pain catastrophizing, intolerance of uncertainty, positive worry beliefs, and cognitive avoidance as potential mechanisms predicting FOC among pregnant women. / Pain catastrophizing and intolerance of uncertainty were the most evident predictors of FOC.
Worldwide prevalence of tocophobia in pregnant women: systematic review and meta-analysis. 4	Ireland, 2017	Systematic review, 33 studies	N/A	To determine the overall pooled prevalence of tokophobia
Fear of childbirth: a neglected dilemma. 5	Finland, 2003	Review	N/A	Preliminary Swedish and Finnish reports demonstrated the results of treatment during pregnancy, when more than half of the women withdrew their request after being able to discuss their anxiety and fear, and vaginal deliveries after treatment were successful.
Fear of childbirth in nulliparous and multiparous women: a population-based analysis of all singleton births in Finland in 1997-2010. 6	Finland, 2014	Cohort study, FOC associations with risk factors and perinatal outcomes	N/A	High and unspecified socioeconomic status, advanced maternal age, and depression are predisposing factors for FOC regardless of parity. Among multiparous women, a previous CS increases vulnerability to FOC. Fear of childbirth is associated with increased rates of cesarean section but does not affect adversely other pregnancy outcomes.
Fear of childbirth in women with normal pregnancy evolution. 7	Finland, 2015	Survey study, 817 women, FOC and previous birth experience	N/A	Fear of childbirth may be seen to some extent in women with a positive birth experience.
Fear of birth in clinical practice: a structured review of current measurement tools. 8	UK, 2018	Review, 46 studies	N/A	The Fear of Birth Scale (FOBS) has been shown to be as effective as the W-DEQ but has the advantage of being short and easy to administer.
Tokophobia: an unreasoning dread of childbirth. A series of 26 cases. 9	UK, 2000	Quantitative analysis interview, 46 women	N/A	Tokophobia is a specific and harrowing condition that needs acknowledging. Close liaison between the obstetrician and the psychiatrist in order to assess the balance between surgical and psychiatric morbidity is imperative with tokophobia.
Tokophobia: A profound dread and avoidance of childbirth (when pathological fear effects the consultation). 10	2007	Book chapter	N/A	N/A
Contents of childbirth-related fear among couples wishing the partner's presence at delivery. 11	Hungary, 1998	Survey, 216 pairs of prospective parents	N/A	Eighty per cent of women and 76% of men felt that the presence of the partner at delivery would have no adverse effect on their future personal relationship.
Fear of childbirth according to parity, gestational age, and obstetric history. 12	Finland, 2008	Survey study	N/A	To examine FOC according to parity, gestational age, and obstetric history. Severe fear of childbirth was more common in nulliparous women, in later pregnancy, and in women with previous cesarean section or VE. Cesarean section as a preferred mode of childbirth was strongly associated with high scores in both the W-DEQ and the VAS.
Identifying women are afraid of giving birth: A comparison of the fear of birth scale with the W-DEQ A in a large Australian cohort. 13	Australia, 2015	To compare the two-item FOBS with the 33-item WDEQ-A in a large cohort of Australian pregnant women, 1,410 women	N/A	Self-report questionnaires during the 2 ^nd^ trimester, including the WDEQ-A and the FOBS. This study supports the use of the FOBS in clinical practice to identify childbirth fear in pregnant women.
Exploring the Fear of Birth Scale in a mixed population of women of childbearing age-A Swedish pilot study. 14	Sweden, 2018	A cross-sectional mixed method study of 179 women	N/A	To explore the FOBS in a mixed sample of women of childbearing age
Fear of childbirth: predictors and temporal changes among nulliparous women in the Danish National Birth Cohort. 15	Denmark, 2008	Cohort of 30 480 healthy nulliparous women with uncomplicated singleton pregnancies.	N/A	To describe the association between FOC and social, demographic, and psychological factors
Fear of childbirth in obstetrically low-risk nulliparous women in Sweden and Denmark. 16	Sweden, 2008	165 women	N/A	The objectives of this study were to compare FOC among Danish and Swedish nulliparous women and to investigate a possible difference in FOC between women who, during pregnancy, had met the midwife who they were subsequently coincidentally allocated to receive labor care from and women who had not previously met the midwife.
Fear of childbirth and duration of labour: a study of 2206 women with intended vaginal delivery. 17	Norway, 2012	Prospective study with 2,206 pregnant women	N/A	Duration of labor was longer in women with FOC than in women without FOC
Cross cultural comparison of levels of childbirth related fear in an Australian and Swedish sample. 18	Australia, 2011	Survey study, 509 women	N/A	To evaluate the range of childbirth-related issues
Higher prevalence of childbirth related fear in foreign born pregnant women–findings from a community sample in Sweden. 19	Sweden, 2015	Cross-sectional prevalence study,	N/A	To investigate the prevalence of CBRF in early pregnancy
Childbirth fear, anxiety, fatigue and sleep deprivation in pregnant women. 20	Canada, 2009	Cross-sectional descriptive survey, 650 women	N/A	To explore women's levels of childbirth fear, sleep deprivation, anxiety, and fatigue and their relationships during the 3 ^rd^ trimester of pregnancy.
Women's lived experience of fear of childbirth. 21	Sweden, 2009	Qualitative study, 8 women	N/A	Four constituents were identified: feeling of danger that threatens and appeals; feeling trapped; feeling like an inferior mother-to-be, and on your own. The essential structure was described as 'to lose oneself as a woman into loneliness'.
A cognitive model of social phobia. 22	USA, 2007	Systematic review, 4 studies	N/A	N/A
Cognitive biases and the emotional disorders. 23	USA, 1992	Book	N/A	Finally, selective associations in fear conditioning are a form of associative bias implicated in the origins of fears and phobias.
Development of the Delivery Fear Scale. 24	Sweden, 2009	Questionnaire development, 135 patients	N/A	This article reviews the development of the DFS to measure fear during labor and delivery.
Pregnant women's thoughts when assessing fear of birth on the Fear of Birth Scale. 25	Sweden, 2016	Qualitative design using semi-structured interviews, 31 pregnant women	N/A	Women thought about aspects that influence their worries and fears and explained the strategies that helped them to cope with their FOC, supporting the use of the FOBS in clinical settings
Causes and outcomes in studies of fear of childbirth: A systematic review. 26	Sweden, 2019	Systematic review, 21 studies	N/A	To summarize the findings of published studies regarding possible causes/predisposing factors and outcomes of FOC for childbearing women.
Secondary fear of childbirth prolongs the time to subsequent delivery. 27	Sweden, 2013	Descriptive, retrospective case-control study, 990 patients	N/A.	The aim of this study was to investigate the time to subsequent delivery and delivery outcome in women with secondary FOC compared with a reference group.
Fear of childbirth and risk for birth complications in nulliparous women in the Danish National Birth Cohort. 28	Denmark, 2009	Prospective cohort study, 25 297 women, computer-assisted telephone interviews	N/A	Risk for emergency cesarean section of women who feared childbirth; risk for dystocia/protracted labor or fetal distress of women who feared childbirth
Fear of childbirth and preference for cesarean delivery among young american women before childbirth. 29	USA, 2015	Online survey with 752 women	N/A	Young women reporting high levels of childbirth fear are nearly four times more likely to prefer a cesarean section. Specific fears, such as worries over the influence of pregnancy and birth on the female body, need to be addressed before pregnancy.
Fear of childbirth and elective caesarean section: a population-based study. 30	Norway, 2015	Cohort study, 1789 women	N/A	Women with FOC may have identifiable vulnerability characteristics, such as poor mental health and poor social support.
Fear of childbirth and risk of caesarean delivery: a cohort study in six European countries. 31	Sweden, 2015	Longitudinal cohort study, 6,422 women	N/A	Having severe FOC increases the risk of elective cesarean delivery, especially among multiparous women. Lack of positive anticipation of the upcoming childbirth seems to be an important dimension of fear associated with cesarean delivery.
The aetiology of post-traumatic stress following childbirth: a meta-analysis and theoretical framework. 32	UK, 2016	Meta-analysis, 50 studies	N/A	Risk factors in birth most strongly associated with PTSD were negative subjective birth experiences (r = 0.59), having an operative birth (assisted vaginal or cesarean, r = 0.48), lack of support (r = -0.38), and dissociation (r = 0.32).

Abbreviations: CBRF, childbirth-related fear; DFS, delivery fear scale; FOC, fear of childbirth; N/A, not applicable; PTSD, post-traumatic stress disorder; WDEQ-A, Wijma Delivery Expectancy/Experience Questionnaire.

## Discussion

### Severe Fear of Childbirth (Tokophobia)

Severe FOC is called tokophobia and is classified within the fourth edition of the Diagnostic and Statistical Manual of Mental Disorders (DSM-IV). 1 2 3 4 5 6 7 8 9 10 11 12 13 14 15 16 17 18 19 20 21 22 23 24 25 26 27 28 29 30 31 32 33 In this situation, FOC gains such a proportion that it will negatively impact the health of a woman, 4 5 turning into a disabling factor that interferes with occupational and domestic functions, as well as with social activities and relationships. 2

Tokophobia is also referred to as an “unreasoning dread of childbirth”; however, no consensus about the definition exists. Many of the articles published so far refer to tokophobia as a severe FOC rather than an irrational dread of childbirth. 6 7 8

Tokophobia is categorized into two forms: primary and secondary. 1 10 Primary tokophobia affects nulliparous women and is the FOC proper. It may result from fears that emerged during adolescence or at the beginning of adulthood, or from stories of experiences told by close persons, or is related to an anxiety disorder. In contrast, secondary tokophobia is the FOC related to a previous birth experience that was negative or traumatic. 2

### Prevalence of Fear of Childbirth

Pregnancy and birth are marked by concerns and fears, observed in up to 80% of habitual-risk pregnant women. 10 There are divergences in the prevalence of FOC and tokophobia between studies, which are mainly due to the lack of consensus regarding the definition of this disease and the variety of assessment instruments used. 11 12 13 A recent study suggested that the global prevalence of FOC can reach 14%, 4 while other studies report a prevalence of 6 to 10%. 14 15 16

### Characteristics of Women with Fear of Childbirth

Nulliparous women are more afraid of childbirth than multiparous women, both in early and in late pregnancy. Furthermore, a more advanced gestational age is associated with a higher level of FOC. A Finnish study involving a sample of 1,400 women demonstrated that pregnant women with < 20 weeks of gestation had lower scores of FOC compared with those with more advanced gestational ages, and this difference was more significant in multiparous than in nulliparous women. 11

Fear of childbirth in women who had 1 previous caesarean section is higher (higher score in the Wijma Delivery Expectancy/Experience Questionnaire [W-DEQ] = 73.2 ± 23.5 [9–150] and higher score in the Visual Analogue Scale [VAS] = 5.1 ± 2.6 [0–10]) than in those with no previous caesarean (W-DEQ = 63.3 ± 20.8 [14–136] and VAS = 2.9 ± 2.5 [0–10]). 11 In a study including Swedish and Australian women, participants with a previous cesarean section reported a negative experience and a higher prevalence of FOC more often than those with a previous vaginal delivery. 17

In addition to parity, gestational age, and previous birth experience, the age and nationality of the woman also seem to influence the FOC. Ternström et al. 19 describe that women < 25 years old had greater FOC than women > 35 years old. The authors also observed greater FOC in foreigners when compared with women born in Sweden.

### Signs and Symptoms

The physiological manifestations of fear include sleep disorders, 19 20 nightmares, 2 5 tachycardia, tension, restlessness, nervousness, and stomach pain. 20 These physiological responses generally interact with cognitive and behavioral aspects, generating anxiety as a response. Examples of cognitive components are automatic negative thoughts 21 , negative expectations and beliefs about yourself, the world, and the future, 22 and specific attention disorders caused by threatening stimuli or situations. 23 34 Regarding behavioral components, the individual starts to avoid situations that are unpleasant and threatening. 1

### Main Causes of Fear of Childbirth

**Fig. 2.**
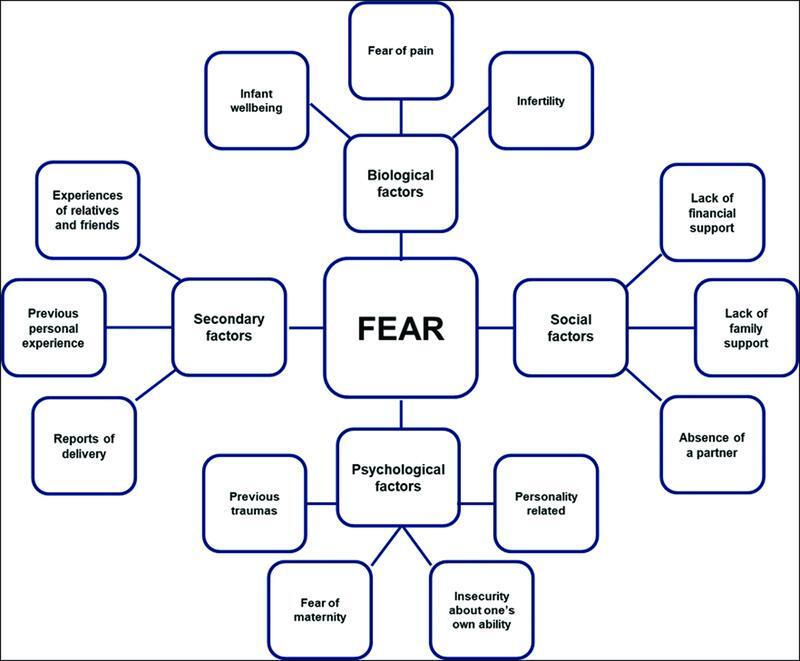
Factors influencing fear of childbirth. **Source:** Adapted from Wijma et al. (2002), 24 Saisto et al. (2003), 5 Ternström et al. (2016), 25 and Dencker et al. (2019) 26

- **Fear of childbirth domains**
  Fear of childbirth in women comprises four domains:
  1. Infant wellbeing.
  2. The labor process ranging from pain, medical interventions, and abnormal evolution of labor to maternal/fetal death.
  3. Personal conditions such as loss of control and distrust of the ability to give birth.
  4. External conditions, especially interaction with the team. 26
Catastrophizing, defined as the tendency to exaggerate the possible negative aspects of pain, 22 24 and the intolerance of uncertainty about childbirth outcomes are considered the most relevant predictors of FOC. 3 Several factors can influence the development of fear of childbirth, including biological factors such as infertility, fear of pain, fear for the wellbeing of the infant, social factors involving the support and environment of the woman, psychological factors related to changes determined by maternity, and factors secondary to previous experiences of the woman and reports of persons close to her ( Figure 2 ). 25
- Fear acquisition and learning
Psychological factors may contribute more strongly to the emergence of FOC and anxiety than demographic and obstetric factors or obstetric history. 3 Fear is acquired through three pathways. 24
  1. Conditioning – when the association that was learned happens. Example: being in a hospital or thinking about childbirth (object or situation) is associated with discomfort (aversive situation).
  2. Indirect exposure – watching someone's delivery.
  3. Indirect exposure through information – reports of another women's delivery.
Indirect exposure and negative experiences can lead to fear acquisition. On the other hand, contact with reports of positive birth experiences can reduce fear. 35
- **Anxiety and depression during pregnancy**
Fear of childbirth during pregnancy has been associated with anxiety, depression, and stress. 36 A study involving 30,480 pregnant women demonstrated a correlation of anxiety and depression during pregnancy with FOC. The authors adjusted for sociodemographic and health factors and obtained an adjusted odds ratio (AOR) of 4.80 (95% confidence interval [CI]: 4.07–5.66). 14
A Finnish study that analyzed 788,317 births found a relative risk of FOC adjusted for depression of 6.35 (95%CI: 5.25–7.68) in nulliparous women and an AOR of 5.47 (95%CI: 4.67–6.41) in multiparous women. 6
- **Preterm birth**
Maternal mental health during pregnancy and its relationship with preterm birth have been studied, but the mechanism whereby maternal mental health triggers physiological events that lead to preterm birth remains unclear. 26 37 38
A systematic review from 2016 demonstrated an association between anxiety during pregnancy and preterm birth. The odds ratio (OR) for antenatal anxiety was 1.70 (95%CI: 1.33–2.18) for preterm birth and 1.67 (95%CI: 1.35–2.07) for spontaneous preterm birth comparing higher levels with lower levels of anxiety. 39 Similarly, another review published in 2018 indicated that anxiety during pregnancy is associated with an increased risk of preterm birth (OR = 1.54; 95%CI: 1.39–1.70; 16 studies) and with an increased risk of spontaneous preterm birth (OR = 1.41; 95%CI: 1.13–1.75). 40
- **Duration of labor**
There is evidence that fear of childbirth leads to an increase in the duration of active labor when the risk is adjusted for socioeconomic variables (AOR = 1.33; 95%CI: 1.11–1.59) in women with FOC. 37 41 A cohort study including 25,297 nulliparous women who were interviewed by phone call twice during pregnancy (early and late pregnancy) showed a higher risk of prolonged labor among women with FOC in both interviews (OR = 1.33; 95%CI: 1.15–1.54). 27
- **Influence of fear of childbirth on the preference of the woman for elective cesarean section**
The World Health Organization (WHO) statement published in 2015 recommends cesarean section rates between 10 and 15% as acceptable. 28 Much higher rates are found in Brazil, reaching 55% within the Brazilian Unified Health System (SUS, in the Portuguese acronym) and 90% in the private sector. Regarding the preferred mode of delivery, cesarean section rates reach 27% among women using the SUS and 44% of those with private health insurance. Factors that explain maternal preference for cesarean delivery in Brazil are maternal convenience and fear of labor pain. 42 These factors are also cited in studies conducted in other countries. 29 43
Since the rates of preference for cesarean delivery in Brazil exceed those reported in other countries, social, economic, and cultural factors may also be related to the choice of delivery mode. Women with private health insurance seem to express more frequently their desire for childbirth, while women using the SUS often do not have this possibility. 44 Another factor that contributes to the high preference for surgical delivery in Brazil is the lack of information of pregnant women about delivery routes so that they can understand the risks and benefits of cesarean and vaginal delivery. 45
It is possible that the diagnosis of tokophobia is the primary cause for requesting cesarean delivery. 5 46 Women who report high levels of FOC are more likely to request a cesarean delivery. 12 47 48 Størksen et al. 30 showed a strong association between FOC and preference for elective cesarean section (OR = 4.6; 95%CI: 2.9–7.3). Situations in which this fear is not treated in a timely manner can increase the chance of a cesarean delivery by up to 5.2 times, 41 and thus lead to a cesarean section without medical indication and exposure of the patient to unnecessary risks. 11 30
Severe FOC may be related directly to an elective or emergency cesarean section in cases of cesarean delivery on request, 30 or indirectly in cases of an increase in uterine contractility and risk of fetal hypoxia triggered by high levels of adrenaline and norepinephrine resulting from exacerbated fear and anxiety. 49
- **Postpartum depression and post-traumatic stress**
Postpartum depression is recognized worldwide as a health condition that can affect between 10 and 15% of women. 31 Furthermore, some pregnancy factors have been associated with the development of post-traumatic stress, especially depression during pregnancy (r = 0.51) and FOC (r = 0.41). 32
  After compiling all this information, we can have an overview of FOC and its implications. The strength of our review is its comprehensive scope, including all major types of clinical investigations, and its thorough search strategy. Also, it brings to light an important topic and the different aspects of its evaluation, since it describes possible influences in fear of childbirth.
  Our scoping review has some limitations. First, there is limited literature about this topic, and this leads to less data to review and evaluate. Also, we did not access the quality of the selected articles, since we had a limited number of studies selected.
  Fear of childbirth is related to an increased risk of adverse obstetric outcomes such as maternal request for cesarean delivery, preterm birth, prolonged labor, postpartum depression, and post-traumatic stress. These evidence highlight the importance of the discussion about FOC on prenatal care and light up an alert for the necessity of strategies for the evaluation and treatment of FOC in the future.

## References

[1] Rondung E Thomtén J Sundin Ö Psychological perspectives on fear of childbirth J Anxiety Disord 2016 44 80 91 10.1016/j.janxdis.2016.10.007 27788373

[2] Nilsson C Hessman E Sjöblom H Dencker A Jangsten E Mollberg M Definitions, measurements and prevalence of fear of childbirth: a systematic review BMC Pregnancy Childbirth 2018 18 01 28 10.1186/s12884-018-1659-7 29329526 PMC5766978

[3] Rondung E Ekdahl J Sundin Ö Potential mechanisms in fear of birth: The role of pain catastrophizing and intolerance of uncertainty Birth 2019 46 01 61 68 10.1111/birt.12368 29954044

[4] O'Connell M A Leahy-Warren P Khashan A S Kenny L C O'Neill S M Worldwide prevalence of tocophobia in pregnant women: systematic review and meta-analysis Acta Obstet Gynecol Scand 2017 96 08 907 920 10.1111/aogs.13138 28369672

[5] Saisto T Halmesmäki E Fear of childbirth: a neglected dilemma Acta Obstet Gynecol Scand 2003 82 03 201 208 12694113

[6] Räisänen S Lehto S M Nielsen H S Gissler M Kramer M R Heinonen S Fear of childbirth in nulliparous and multiparous women: a population-based analysis of all singleton births in Finland in 1997-2010 BJOG 2014 121 08 965 970 10.1111/1471-0528.12599 24494605

[7] Aksoy A N Ozkan H Gundogdu G Fear of childbirth in women with normal pregnancy evolution Clin Exp Obstet Gynecol 2015 42 02 179 183 26054114

[8] Richens Y Smith D M Lavender D T Fear of birth in clinical practice: A structured review of current measurement tools Sex Reprod Healthc 2018 16 98 112 10.1016/j.srhc.2018.02.010 29804785

[9] Hofberg K Brockington I Tokophobia: an unreasoning dread of childbirth. A series of 26 cases Br J Psychiatry 2000 176 83 85 10.1192/bjp.176.1.83 10789333

[10] Hofberg K Ward M Tokophobia tokophobia: a profound dread and avoidance of childbirth (when pathological fear effects the consultation) London Springer 2007 165 72

[11] Szeverényi P Póka R Hetey M Török Z Contents of childbirth-related fear among couples wishing the partner's presence at delivery J Psychosom Obstet Gynaecol 1998 19 01 38 43 10.3109/01674829809044219 9575467

[12] Rouhe H Salmela-Aro K Halmesmäki E Saisto T Fear of childbirth according to parity, gestational age, and obstetric history BJOG 2009 116 01 67 73 10.1111/j.1471-0528.2008.02002.x 19055652

[13] Haines H M Pallant J F Fenwick J Gamble J Creedy D K Toohill J Identifying women who are afraid of giving birth: A comparison of the fear of birth scale with the WDEQ-A in a large Australian cohort Sex Reprod Healthc 2015 6 04 204 210 10.1016/j.srhc.2015.05.002 26614602

[14] Hildingsson I Rubertsson C Karlström A Haines H Exploring the Fear of Birth Scale in a mixed population of women of childbearing age-A Swedish pilot study Women Birth 2018 31 05 407 413 10.1016/j.wombi.2017.12.005 29249331

[15] Danish National Birth Cohort Laursen M Hedegaard M Johansen C Fear of childbirth: predictors and temporal changes among nulliparous women in the Danish National Birth Cohort BJOG 2008 115 03 354 360 10.1111/j.1471-0528.2007.01583.x 18190372

[16] Kjærgaard H Wijma K Dykes A Alehagen S Fear of childbirth in obstetrically low-risk nulliparous women in Sweden and Denmark J Reprod Infant Psychol 2008 26 04 340 350 10.1080/02646830802408498

[17] Adams S S Eberhard-Gran M Eskild A Fear of childbirth and duration of labour: a study of 2206 women with intended vaginal delivery BJOG 2012 119 10 1238 1246 10.1111/j.1471-0528.2012.03433.x 22734617

[18] Haines H Pallant J F Karlström A Hildingsson I Cross-cultural comparison of levels of childbirth-related fear in an Australian and Swedish sample Midwifery 2011 27 04 560 567 10.1016/j.midw.2010.05.004 20598787

[19] Ternström E Hildingsson I Haines H Rubertsson C Higher prevalence of childbirth related fear in foreign born pregnant women–findings from a community sample in Sweden Midwifery 2015 31 04 445 450 10.1016/j.midw.2014.11.011 25529841

[20] Hall W A Hauck Y L Carty E M Hutton E K Fenwick J Stoll K Childbirth fear, anxiety, fatigue, and sleep deprivation in pregnant women J Obstet Gynecol Neonatal Nurs 2009 38 05 567 576 10.1111/j.1552-6909.2009.01054.x 19883478

[21] Nilsson C Lundgren I Women's lived experience of fear of childbirth Midwifery 2009 25 02 e1 e9 10.1016/j.midw.2007.01.017 17512645

[22] Clark D M Wells A A cognitive model of social phobia New York Guilford 1995 69 93

[23] Mineka S Sutton S K Cognitive biases and the emotional disorders Psychol Sci 1992 3 01 65 69 10.1111/j.1467-9280.1992.tb00260.x

[24] Wijma K Alehagen S Wijma B Development of the Delivery Fear Scale J Psychosom Obstet Gynaecol 2002 23 02 97 107 10.3109/01674820209042791 12189903

[25] Ternström E Hildingsson I Haines H Rubertsson C Pregnant women's thoughts when assessing fear of birth on the Fear of Birth Scale Women Birth 2016 29 03 e44 e49 10.1016/j.wombi.2015.11.009 26710973

[26] Dencker A Nilsson C Begley C Causes and outcomes in studies of fear of childbirth: A systematic review Women Birth 2019 32 02 99 111 10.1016/j.wombi.2018.07.004 30115515

[27] Sydsjö G Angerbjörn L Palmquist S Bladh M Sydsjö A Josefsson A Secondary fear of childbirth prolongs the time to subsequent delivery Acta Obstet Gynecol Scand 2013 92 02 210 214 10.1111/aogs.12034 23066797

[28] Laursen M Johansen C Hedegaard M Fear of childbirth and risk for birth complications in nulliparous women in the Danish National Birth Cohort BJOG 2009 116 10 1350 1355 10.1111/j.1471-0528.2009.02250.x 19538412

[29] Stoll K Edmonds J K Hall W A Fear of childbirth and preference for cesarean delivery among young american women before childbirth: a survey study Birth 2015 42 03 270 276 10.1111/birt.12178 26104997

[30] Størksen H T Garthus-Niegel S Adams S S Vangen S Eberhard-Gran M Fear of childbirth and elective caesarean section: a population-based study BMC Pregnancy Childbirth 2015 15 221 10.1186/s12884-015-0655-4 26382746 PMC4573308

[31] Bidens Group Ryding E L Lukasse M Parys A S Wangel A-M Karro H Kristjansdottir H Fear of childbirth and risk of cesarean delivery: a cohort study in six European countries Birth 2015 42 01 48 55 10.1111/birt.12147 25676793

[32] Ayers S Bond R Bertullies S Wijma K The aetiology of post-traumatic stress following childbirth: a meta-analysis and theoretical framework Psychol Med 2016 46 06 1121 1134 10.1017/S0033291715002706 26878223

[33] Bell C C DSM-IV: Diagnostic and Statistical Manual of Mental Disorders JAMA 1994 272 10 828 829 10.1001/jama.1994.03520100096046

[34] Foa E B Huppert J D Cahill S P Emotional processing theory: an update New York Guilford 2006 3 24

[35] Rachman S The conditioning theory of fear-acquisition: a critical examination Behav Res Ther 1977 15 05 375 387 10.1016/0005-7967(77)90041-9 612338

[36] Bandura A The social learning theory of aggression London Routledge 2019 141 58

[37] Wadhwa P D Culhane J F Rauh V Barve S S Stress and preterm birth: neuroendocrine, immune/inflammatory, and vascular mechanisms Matern Child Health J 2001 5 02 119 125 10.1023/a:1011353216619 11573837

[38] Ruiz R J Fullerton J Dudley D J The interrelationship of maternal stress, endocrine factors and inflammation on gestational length Obstet Gynecol Surv 2003 58 06 415 428 10.1097/01.OGX.0000071160.26072.DE 12775946

[39] Goldenberg R L Culhane J F Iams J D Romero R Epidemiology and causes of preterm birth Lancet 2008 371(9606):75 84 10.1016/S0140-6736(08)60074-4 18177778 PMC7134569

[40] Rose M S Pana G Premji S Prenatal maternal anxiety as a risk factor for preterm birth and the effects of heterogeneity on this relationship: a systematic review and meta-analysis BioMed Res Int 2016 2016 8.312158E6 10.1155/2016/8312158 PMC4889802 27298829

[41] Grigoriadis S Graves L Peer M Mamisashvili L Tomlinson G Vigod S N Maternal anxiety during pregnancy and the association with adverse perinatal outcomes: systematic review and meta-analysis J Clin Psychiatry 2018 79 05 17r12011 10.4088/JCP.17r12011 30192449

[42] World Health Organization Global report on diabetes [Internet] Geneva World Health Organization 2016[cited 2021 Sep 14]. Available from:https://apps.who.int/iris/handle/10665/204871

[43] Reiter M Betrán A P Marques F K Torloni M R Systematic review and meta-analysis of studies on delivery preferences in Brazil Int J Gynaecol Obstet 2018 143 01 24 31 10.1002/ijgo.12570 29920679

[44] Mazzoni A Althabe F Gutierrez L Gibbons L Liu N H Bonotti A M Women's preferences and mode of delivery in public and private hospitals: a prospective cohort study BMC Pregnancy Childbirth 2016 16 34 10.1186/s12884-016-0824-0 26857448 PMC4746891

[45] Rattner D Moura E C Nascimentos no Brasil: associação do tipo de parto com variáveis temporais e sociodemográficas. Rev Bras Saúde Matern Infant 2016 16 01 39 47 10.1590/1806-93042016000100005

[46] Domingues R M Dias M A Nakamura-Pereira M Torres J A d'Orsi E Pereira A PE Process of decision-making regarding the mode of birth in Brazil: from the initial preference of women to the final mode of birth Cad Saude Publica 2014 30 01 S1 S16 10.1590/0102-311X00105113 25167169

[47] Wax J R Cartin A Pinette M G Blackstone J Patient choice cesarean: an evidence-based review Obstet Gynecol Surv 2004 59 08 601 616 10.1097/01.ogx.0000133942.76239.57 15277895

[48] Dweik D Girasek E Töreki A Mészáros G Pál A Women's antenatal preferences for delivery route in a setting with high cesarean section rates and a medically dominated maternity system Acta Obstet Gynecol Scand 2014 93 04 408 415 10.1111/aogs.12353 24575805

[49] Wiklund I Edman G Ryding E L Andolf E Expectation and experiences of childbirth in primiparae with caesarean section BJOG 2008 115 03 324 331 10.1111/j.1471-0528.2007.01564.x 18190368

